# Editorial: Advances in understanding and managing congenital heart disease: from bench to bedside

**DOI:** 10.3389/fped.2025.1628126

**Published:** 2025-08-14

**Authors:** Corina Maria Vasile, Xavier Iriart

**Affiliations:** Department of Pediatric and Adult Congenital Cardiology, Bordeaux University Hospital, Pessac, France

**Keywords:** congenital heart disease, cardiac, artificial intelligence, cardiac defects, pediatric cardiology, interventional cardiology

**Editorial on the Research Topic**
Advances in understanding and managing congenital heart disease: from bench to bedside

Significant advancements in medical and surgical interventions have greatly improved survival rates, enabling more patients with congenital heart disease (CHD) to reach adulthood (1, 2). This development highlights the importance of ongoing innovation in diagnostic methods, therapeutic strategies, and long-term management approaches. The synergy between basic scientific research and clinical practice—encompassed in the “bench-to-bedside” paradigm—has been crucial in translating molecular and genetic discoveries into meaningful clinical applications, thereby enhancing patient outcomes and quality of life. This Research Topic, “*Advances in Understanding and Managing Congenital Heart Disease: From Bench to Bedside*,” brings together contemporary studies that connect foundational research with clinical implementation, tackling the diverse challenges associated with congenital heart disease (CHD).

Understanding the epidemiological trends of CHD is crucial for developing effective healthcare strategies. Deng et al. provide a global perspective on CHD by utilizing data from the Global Burden of Disease Study (1990–2021). Their findings highlight a substantial 54.58% global decrease in CHD-associated infant mortality between 1990 and 2021. Despite this significant progress, pronounced disparities remain, with regions of low socioeconomic development continuing to experience the highest prevalence, mortality rates, and disability burdens. These results underscore the critical necessity for better healthcare policies, expanded screening programs, and improved access to surgical services interventions.

Timely and accurate diagnosis is fundamental to improving CHD management. Liu et al. reviewed recent advancements in artificial intelligence (AI) for the prenatal diagnosis of congenital heart disease (CHD), emphasizing that AI significantly improves diagnostic efficiency, accuracy, and clinician confidence, particularly through the application of deep learning techniques to fetal echocardiography. However, they highlighted ongoing challenges, such as variability in fetal imaging (volatility), inadequate multidimensional training data (insufficiency), and fragmented decision-making processes (independence), which currently limit broader clinical adoption. The authors suggest addressing these challenges through the use of more comprehensive datasets and optimized clinical decision-making pathways to enhance the clinical effectiveness of AI in prenatal CHD management.

Meanwhile, Lagerstrand et al. demonstrated that partial volume correction significantly improved the accuracy of pulmonary perfusion measurements obtained by 4D flow cardiac magnetic resonance imaging (CMR) in pediatric patients with congenital heart disease. Before the correction, differences in pulmonary perfusion ratio compared to lung scintigraphy reached up to 178%, particularly in patients with smaller pulmonary arteries. After applying partial volume correction, these differences were markedly reduced to less than 21%, confirming 4D flow CMR as a reliable, radiation-free alternative for evaluating pulmonary perfusion in children with congenital heart disease (CHD).

Arrhythmias in CHD patients pose significant clinical challenges, particularly as more patients survive into adulthood. Paja et al. demonstrated the effectiveness of remote magnetic navigation, combined with advanced electroanatomical mapping, in successfully ablating multiple accessory pathways in a young adult with complex congenital heart disease and single-ventricle physiology. Despite challenging anatomical features due to multiple corrective surgeries, the procedure successfully eliminated two accessory pathways, and the patient remained free of arrhythmia recurrence for more than two years post-ablation without the need for ongoing antiarrhythmic therapy.

Surgical intervention remains a cornerstone in CHD management, with ongoing improvements in techniques and perioperative care. Bulescu et al. concluded that the use of Celsior® cardioplegia in pediatric arterial switch surgery is both effective and safe, providing comparable myocardial protection to the traditional St Thomas® solution. Importantly, the Celsior® solution significantly reduced the need for postoperative ECMO support (4.9% vs. 24.7%, *p* < 0.001) and shortened ICU stays (4.6 days vs. 8.7 days, *p* < 0.001). While these results indicate potential clinical benefits, the authors emphasize the need for prospective randomized trials to confirm these findings definitively.

Barbier et al. also investigated the outcomes of complex aortic valve repair in patients with congenital heart disease. They reported an excellent postoperative survival rate of 92.85% and a freedom from reoperation of 85.7% at three years. Echocardiographic follow-up demonstrated a significant improvement in maximal and mean aortic valve gradients (*p* = 0.02), confirming the efficacy and durability of complex aortic valve repair as an effective primary surgical option in patients with congenital heart disease.

Beyond survival, optimizing neurodevelopmental outcomes in patients with CHD is a growing priority. Tran et al. found significant differences in preoperative cerebral oxygenation (rcSO2) and cerebral fractional tissue oxygen extraction (FTOE) between neonates with congenital heart disease (CHD) and healthy controls. Neonates with CHD had lower rcSO2 values (67% vs. 79%, *p* < 0.001) and higher FTOE values (0.27 vs. 0.19, *p* < 0.001). Interestingly, while increased cerebral oxygenation correlated with improved neurobehavioral outcomes in neonates with CHD, it was significantly associated with poorer outcomes in healthy controls (interaction *p* = 0.004). These findings highlight a critical range for cerebral oxygenation in neonates, suggesting that extremes in cerebral oxygenation levels—either too low or too high—may affect neurodevelopment differently depending on the status of CHD.

Effective decision-making is a vital component of high-quality pediatric cardiac care. Padovani et al. highlight the influence of cognitive biases in clinical reasoning. This review highlights how cognitive biases, including anchoring, confirmation, and availability biases, frequently influence clinical decision-making in pediatric cardiology, potentially leading to diagnostic errors. It emphasizes recognizing and mitigating these biases through structured training to enhance clinical judgment and improve patient outcomes in pediatric cardiac care.

The studies presented in this Research Topic highlight significant advancements in CHD research and clinical practice. Covering epidemiological research, AI-enhanced diagnostics, surgical innovations, and neurodevelopmental aspects, these findings highlight the crucial role of a multidisciplinary approach in improving outcomes for CHD patients.

This Research Topic fosters collaboration between scientists and clinicians, bridging the gap between laboratory research and clinical practice, which ultimately drives innovation and improves the lives of those affected by congenital heart disease.
